# Prevalence, associated factors, and machine learning classification of low back pain among postmenopausal women attending rehabilitation and physiotherapy facilities in Bangladesh

**DOI:** 10.1371/journal.pone.0355864

**Published:** 2026-09-21

**Authors:** Md. Faruqul Islam, Md. Omar Faruque, Md. Alauddin, Md. Omar Faruk, Zakia Rahman, Zahid Bin Sultan Nahid, Ahsan Habib Bulbul, Md Emran Hasan, Maysa Mohamed Rabea Abdelall, Firoj Al-Mamun, Mohammed A. Mamun

**Affiliations:** 1 Department of Nutrition and Food Technology, Jashore University of Science and Technology, Jashore, Bangladesh; 2 Department of Physiotherapy, Saic College of Medical Science and Technology, Dhaka, Bangladesh; 3 CHINTA Research Bangladesh, Dhaka, Bangladesh; 4 Department of Physiotherapy, Dhaka College of Physiotherapy, Dhaka, Bangladesh; 5 School of Computer Science and Engineering, Nanjing University of Science and Technology, Nanjing, China; 6 Department of Physical Sport Sciences, College of Sport Sciences and Physical Activity, Princess Nourah bint Abdulrahman University, Riyadh, Saudi Arabia; 7 Department of Public Health, University of South Asia, Dhaka, Bangladesh; Bangladesh University of Engineering and Technology, BANGLADESH

## Abstract

**Background:**

Low back pain (LBP) is a leading cause of disability worldwide and disproportionately affects women, particularly after menopause. However, evidence on the prevalence and associated factors of LBP among postmenopausal women in low- and middle-income countries remains limited. This study aimed to estimate the past-year prevalence of LBP among postmenopausal women attending selected rehabilitation and physiotherapy facilities in Bangladesh, examine factors associated with LBP, and evaluate the internal classification performance of machine learning models.

**Methods:**

A clinic-based cross-sectional study was conducted among 566 postmenopausal women attending two specialized rehabilitation centers in Bangladesh. Sociodemographic, clinical, lifestyle, and socioeconomic data were collected using structured questionnaires. Multivariable logistic regression was used to estimate adjusted associations with LBP. Machine learning models (Logistic Regression, Support Vector Machine with radial basis function (SVM-RBF), Random Forest, Bernoulli Naïve Bayes, and Extreme Gradient Boosting) were developed to classify LBP. Model development used nested cross-validation, and final discriminative performance was evaluated using a held-out test set. Model interpretability was assessed using SHapley Additive exPlanations.

**Results:**

The prevalence of past-year LBP among the postmenopausal women was 46.6%. Multivariable analysis showed lower odds of LBP among women aged 51–57 years than those aged 43–50 years (AOR = 0.50, 95% CI: 0.31–0.81), and higher odds among housewives (AOR = 2.20, 95% CI: 1.33–3.64), women with hypertension (AOR = 1.60, 95% CI: 1.09–2.35), respiratory disease (AOR = 2.46, 95% CI: 1.28–4.71), and selected socioeconomic groups, while heart disease and BMI category were not clearly associated after adjustment. Among the evaluated machine learning models, the SVM-RBF classifier achieved the highest accuracy during nested cross-validation (0.772), while random forest achieved comparable accuracy (0.768) and the highest F1-score (0.744). On the held-out test set, SVM-RBF achieved the highest ROC-AUC (0.890), followed by random forest (0.885). SHAP analysis identified sunlight exposure, hypertension, and socioeconomic status as the most influential contributors to model classification.

**Conclusions:**

LBP is commonly reported among postmenopausal women seeking rehabilitation and physiotherapy care in Bangladesh and is associated with multiple social, occupational, and health-related factors. Machine learning models demonstrated relatively strong internal classification performance; however, external validation is required before broader clinical use can be considered. Longitudinal community- and clinic-based studies are needed to clarify temporal relationships and assess the generalizability of these findings.

## 1. Introduction

Low back pain (LBP) is characterized by pain, muscle tension, or stiffness located below the costal margin and above the inferior gluteal folds, with or without leg pain [1–3]. As one of the most prevalent musculoskeletal disorders globally, LBP remains a leading contributor to long-term disability and restrictions in daily activities across diverse populations [2,4]. Beyond its direct health impact, LBP imposes substantial social and economic burdens ranging from increased healthcare demands to significant losses in workplace productivity and overall quality of life [1,5]. Epidemiological data consistently show that women are disproportionately affected, experiencing greater pain severity, chronicity, and disability compared to men [5,6]. Recent global estimates indicate that approximately 619 million people were affected by LBP in 2020, with this number projected to rise dramatically to 843 million by 2050 [7,8]. The prevalence of LBP steadily increases with age, reaching its highest levels among older adults and making it the leading cause of years lived with disability (YLDs) worldwide [1,7]. While both men and women are impacted, women, particularly in the peri- and postmenopausal years, may experience a greater burden and more severe impact, with the gender gap in prevalence and burden widening after menopause [6].

A growing body of research highlights the substantial burden of LBP worldwide. International pooled data indicate that LBP affects a substantial proportion of the general population, with meta-analyses reporting lifetime, annual, and point prevalence rates of 66%, 51%, and 48% in India [9] and 47%, 57%, and 39% in Africa, respectively [10]. Systematic reviews and multi-country studies further show that the burden of LBP increases with age, significantly impacting functional mobility, quality of life, and work productivity [1,3]. Although both sexes are affected, women consistently report higher prevalence and severity, particularly after midlife due to hormonal, biological, and social factors [5,6]. Among women, the postmenopausal period marks a time of heightened musculoskeletal vulnerability, with hormonal changes compounding this burden. For example, Ogwumike et al. [11] reported that 46.8% of postmenopausal women in Nigeria reported back symptoms during the previous 12 months, while the rate of LBP can be as high as 90.2% as reported in Turkish postmenopausal women [12]. In Northern India, 77.8% of postmenopausal women reported LBP, with being 6–10 years rather than 1–5 years after menopause associated with approximately fourfold higher adjusted odds of severe pain [13]. Even among postmenopausal women participating in an Italian trial, approximately one-third reported LBP at baseline [14].

In Bangladesh, Majumder et al. [15] conducted a large-scale, community-based survey using a nationally representative and stratified sample, and found a weighted prevalence of LBP of 18.5% among adults (aged ≥18 years). The age-standardized prevalence was higher in women (27.2%) than in men (14.0%), and age-specific prevalence increased with age to 27.8% among adults aged 55 years and older [15]. Similarly, a recent cross-sectional study among bicycle rickshaw pullers in Dhaka reported a self-reported LBP prevalence of 33%, with higher rates among those exposed to heavy lifting and stressful working conditions [16]. Further regional evidence from Bishwajit et al. [17], who analyzed World Health Survey data from five South Asian countries, reported that the prevalence of self-reported back pain during the previous 30 days among adults aged 50 years and older varied across the included countries, with estimates of 64.8% in Bangladesh, 19.8% in India, 69.5% in Nepal, 40.6% in Pakistan, and 36.2% in Sri Lanka. These findings highlight the burden of LBP in Bangladesh and the need for research using clearly defined populations, settings, and outcome measures. However, prevalence estimates from community-based surveys, occupational groups, and clinical populations should not be interpreted as directly comparable because they may differ in sampling methods, outcome definitions, recall periods, and healthcare-seeking characteristics.

The increased prevalence of LBP among postmenopausal women is believed to be closely linked to hormonal changes, particularly the decline in estrogen following menopause, which accelerates intervertebral disc degeneration and heightens susceptibility to spondylolisthesis and facet joint osteoarthritis [6]. Nonetheless, LBP is recognized as a multifactorial disorder with a complex and interwoven etiology, shaped by a wide array of biological, psychological, and environmental factors [3]. Socioeconomic status, occupational exposures, comorbidities such as heart or respiratory disease, and psychosocial stressors may be associated with the occurrence, chronicity, and severity of LBP [3,18]. The biopsychosocial model of LBP highlights how these factors interact to influence pain perception, coping behaviors, and disability outcomes [1,3]. As a lower-middle-income country, Bangladesh faces unique challenges, including socio-economic adversity, limited healthcare resources, and a high burden of chronic conditions among women, all of which may shape the burden and consequences of LBP in postmenopausal women. Despite the significant public health implications, evidence specifically focused on postmenopausal women seeking rehabilitation or physiotherapy care in Bangladesh remains limited.

In recent years, machine learning (ML) techniques have increasingly been applied in epidemiological and clinical research to analyze complex health datasets and explore classification patterns. Unlike traditional statistical approaches, ML algorithms can capture nonlinear relationships and interactions among multiple variables, potentially complementing conventional regression-based analyses of multifactorial health outcomes. A scoping review identified 53 studies applying machine learning methods in chronic pain research, many of which focused on the classification and prediction of pain conditions using clinical, behavioral, and demographic data [19]. Similarly, recent evidence indicates that artificial intelligence approaches are being used to predict clinical outcomes, analyze clinical records, identify patient subgroups, and model complex biological data in chronic pain research, thereby supporting further investigation of complex clinical patterns and pain-related outcomes [20]. Despite these advances, the application of ML approaches to investigate low back pain among postmenopausal women, particularly in low- and middle-income settings, remains limited. Integrating ML techniques with conventional statistical analyses may therefore provide additional insights into association patterns and internally derived classification features for LBP within specific clinical populations. However, any model intended for clinical application requires external validation, calibration, and assessment of clinical utility.

Given this context, evidence remains limited regarding LBP among postmenopausal women attending specialized rehabilitation centers in Bangladesh, a healthcare-seeking group whose symptom burden may differ from that of women in the wider community. The present study aims to (1) estimate the past-year prevalence of LBP among postmenopausal women attending two specialized rehabilitation centers, (2) identify sociodemographic, clinical, and lifestyle factors associated with LBP, and (3) evaluate the internal classification performance of several machine learning models for LBP using demographic and health-related variables. By combining conventional statistical analysis with machine learning approaches, this study seeks to provide deeper insights into the association patterns and classification performance of LBP within this specific healthcare-seeking population.

## 2. Methods

### 2.1. Study design, setting, and participants

This was a clinic-based cross-sectional study conducted among postmenopausal women attending selected rehabilitation and physiotherapy centers in Bangladesh. Data were collected from December 2022 to December 2023 at the Centre for the Rehabilitation of the Paralyzed (CRP), including its Savar and Dhaka/Mirpur facilities, and the Unique Pain and Paralysis Centre (UPPC), Dhaka/Mirpur. Participants included postmenopausal women who came to these centers for assessment or treatment with general musculoskeletal complaints.

This study included women 40 years or older who reported natural menopause, defined as the permanent cessation of menstruation for at least 12 consecutive months, had an intact uterus with at least one ovary, and reported musculoskeletal disorders or musculoskeletal problems, with LBP status subsequently identified using the musculoskeletal symptom assessment described below. Women were excluded if they were under 40, unwilling to participate, currently pregnant or breastfeeding, diagnosed with polycystic ovary syndrome, undergoing chemotherapy, had surgically induced menopause (e.g., hysterectomy), or were medically unstable (e.g., HIV/AIDS). Additionally, those with neurological disorders, cognitive or hearing impairments, or communication difficulties were also excluded.

### 2.2. Sample size and sampling

Sample-size adequacy for this cross-sectional analysis was evaluated using the single-proportion formula, n = Z²p(1-p)/d². Because no directly comparable prevalence estimate was available for LBP among postmenopausal women attending rehabilitation or physiotherapy centers in Bangladesh, the expected prevalence was set at 50% to provide a conservative maximum sample-size estimate. Using a 95% confidence level, a 5% margin of error, and p = 0.50, the minimum required sample size was 385 participants.

Participants were recruited using non-probability purposive sampling from the selected rehabilitation and physiotherapy centers. Eligible women attending the study settings during the data-collection period were approached according to the prespecified inclusion and exclusion criteria. This sampling approach enabled focused recruitment of postmenopausal women with musculoskeletal complaints; however, because recruitment occurred in clinical settings rather than through community-based probability sampling, the findings should be interpreted as applying primarily to a healthcare-seeking population. A total of 576 participants were recruited, exceeding the minimum required sample size. After data cleaning, 10 records were excluded because of incomplete data, failure to meet the eligibility criteria, or duplicate entries, leaving 566 valid records for the present analysis.

### 2.3. Questionnaire development, translation, and pilot testing

A structured questionnaire was developed in English based on the study objectives and relevant literature. The questionnaire incorporated standardized tools and structured sections. The questionnaire was translated into Bangla using forward and backward translation procedures. Two medically trained bilingual translators independently translated the English questionnaire into Bangla, and another medically trained bilingual translator performed backward translation to check whether the original meaning was retained. The translated questionnaire was pilot-tested among 60 postmenopausal women who met the eligibility criteria. Based on the pilot findings, face-to-face interviews were selected instead of self-administered questionnaires to minimize misunderstanding and improve response quality.

### 2.4. Data collection procedure and quality control

Interviews were conducted face-to-face in Bangla in a private setting within the participating healthcare facilities. Before data collection, all data collectors received orientation on the study objectives, questionnaire content, ethical considerations, informed-consent procedures, and standardized interviewing techniques to ensure consistency across sites. Questions were explained to participants whenever clarification was needed, while maintaining neutrality to avoid influencing responses. Data were collected by trained health professionals, including physiotherapists working in the study settings, with support from trained nutrition and food science personnel for dietary assessments and anthropometric measurements. Throughout the data-collection period, the principal investigator provided regular supervision and conducted spot checks to ensure adherence to study protocols. Completed questionnaires were reviewed daily by the principal investigator and research team for completeness, consistency, and accuracy, and any discrepancies or missing information were addressed whenever possible before data entry and analysis.

### 2.5. Measures

#### 2.5.1. Sociodemographic factors.

Participants reported their age, marital status, current occupation, educational attainment, administrative division of residence, and residential area (urban or rural). Information about family structure was also collected, including whether they lived in nuclear or extended households. The socioeconomic status of participants was calculated using the Modified Kuppuswamy Scale [21], which categorizes socioeconomic position using education, occupation, and total family income. For the present analysis, socioeconomic status was grouped into upper, upper-middle, lower-middle, and upper-lower categories according to the scale classification used in the original study.

#### 2.5.2. Health-related and lifestyle factors.

The questionnaire contained structured items on smoking, sunlight exposure, weekly exercise, hormone therapy, and calcium supplementation. Sunlight exposure was assessed by asking whether participants stayed in sunlight every day and was coded as yes or no. Weekly exercise was assessed by asking participants how often they exercised in a week and was categorized as not at all, 1–2 times a week, 3–4 times a week, or >4 times a week. Hormone therapy and calcium supplementation were recorded based on participants’ self-reported use. Comorbid conditions were assessed by asking whether participants were suffering from any disease other than musculoskeletal problems, with response options including hypertension, diabetes, respiratory disease, heart disease, other disease, and no disease. Participants were also asked whether they had ever had an abortion. All health-related and lifestyle variables were self-reported unless otherwise specified.

#### 2.5.3. Body mass index (BMI).

Using measured height and weight data, we calculated BMI as weight in kilograms divided by height in meters squared (kg/m²). Body weight was measured using a digital weight scale after participants removed shoes, heavy clothing, and heavy items from their pockets. The scale was zeroed before measurement, and participants were asked to stand still while the measurement was recorded. Standing height was measured using a measuring tape against a flat wall and floor surface after participants removed shoes, headwear, and bulky clothing. Participants stood upright with heels against the wall and eyes facing forward, and height was recorded to the nearest available measurement, following the original study procedure. After calculating the BMI for each participant, the results were categorized into the World Health Organization (WHO) extended classification, which includes: mild thinness (17.00–18.49 kg/m²), normal weight (18.50–24.99 kg/m²), overweight (25.00–29.99 kg/m²), obese class I (30.00–34.99 kg/m²), and obese class II (35.00–39.99 kg/m²).

#### 2.5.4. Low back pain assessment.

Low back pain (LBP) was assessed using the musculoskeletal complaint section of the questionnaire, adapted from the Standardized Nordic Musculoskeletal Questionnaire. First, participants were asked whether they had experienced musculoskeletal trouble, such as ache, pain, discomfort, or numbness, during the previous 12 months. Participants who reported symptoms were then asked to identify the affected body region from a list that included the neck, shoulder, upper back, elbow, wrist/hand, low back, hip/thigh, knee, and ankle/feet. For the present analysis, participants who selected the low-back region were classified as having LBP, whereas those who did not select the low-back region were classified as not having LBP. Participants reporting low-back symptoms were also asked whether they had any reported diagnosed low-back musculoskeletal condition, such as prolapsed lumbar intervertebral disc, spondylolisthesis, lumbar spondylosis, piriformis syndrome, sacroiliac joint dysfunction, or another specified condition. This information was collected to describe reported low-back conditions where available; however, the primary LBP classification used in this analysis was based on structured self-reported low-back symptoms during the previous 12 months.

### 2.6. Ethics statement

This study received ethical clearance from the Institutional Review Board of Jashore University of Science and Technology (JUST) and the Ethical Review Board of the Centre for the Rehabilitation of the Paralyzed (CRP) [Reference Number: CRP-RZE-0401–0411]. Relevant documents, including the study proposal, informed consent form, and questionnaires in both English and Bangla, were submitted for review. The study followed ethical guidelines set by the World Health Organization (WHO) and the revised Declaration of Helsinki (2013). Written permission for data collection was also obtained from study site authorities. Participants were informed about the study’s purpose, procedures, and their rights. After receiving a verbal explanation of the study, participants provided written informed consent using the study consent form. Participation was voluntary, and respondents could withdraw at any time. Confidentiality and privacy were strictly maintained, and no form of harm or coercion occurred. No incentives were provided for participation.

### 2.7. Statistical analysis

Data were analyzed using R statistical software (version 4.5.2). The primary outcome was past-year low back pain, coded as 1 = yes and 0 = no. Descriptive statistics were used to summarize participant characteristics. Categorical variables were presented as frequencies and percentages. The prevalence of previous-12-month LBP was calculated as the proportion of participants classified as having LBP among the total analytical sample. Bivariate analyses used observations with available data on the variables being compared. Crude and adjusted logistic regression analyses used complete cases for the outcome and all included covariates.

Bivariate associations between participant characteristics and past-year LBP were examined using chi-square tests. Fisher’s exact test was used when expected cell counts were small; for larger contingency tables with sparse expected counts, Fisher’s exact test with simulated p-values was applied. Row percentages were reported to show the proportion of participants with and without LBP within each category. Cramer’s V was calculated as a measure of association strength for bivariate comparisons.

Binary logistic regression was used to estimate crude and adjusted odds ratios (ORs) with 95% confidence intervals (CIs) for factors associated with past-year LBP. Original variable categories were retained for descriptive and bivariate analyses. For adjusted regression, selected variables with sparse categories were collapsed to improve model stability. Specifically, division was grouped as Dhaka versus outside Dhaka; age was grouped as 43–50, 51–57, 58–65, and 66 years or above; education was grouped as graduate or above, secondary or higher secondary, and up to primary; marital status was grouped as married versus not currently married; occupation was grouped as housewife versus other; family structure was grouped as nuclear family versus joint family or living alone; and BMI was grouped as normal/thinness, overweight, and obese. Smoking was summarized descriptively but was not included in the adjusted logistic regression model because of sparse exposure counts. The full coding approach used for descriptive and adjusted regression analyses is provided in supplementary material (S1 Table in **S1 File**). The adjusted logistic regression model included division, residence, age group, education, marital status, religion, occupation, family structure, sunlight exposure, hormone therapy, calcium supplementation, hypertension, diabetes, respiratory disease, heart disease, weekly exercise, abortion history, BMI category, and socioeconomic status. Category-specific p-values were reported for individual non-reference categories, and overall p-values for multi-category variables were obtained using likelihood-ratio tests. Multicollinearity was assessed using generalized variance inflation factors, with adjusted GVIF values used for multi-level categorical predictors. Model diagnostics included convergence status, Akaike information criterion, Bayesian information criterion, McFadden pseudo-R^2^, area under the receiver operating characteristic curve, and the Hosmer–Lemeshow goodness-of-fit test. Model diagnostics and multicollinearity results are provided in **S2 and S3 Tables in S1 File**. All tests were two-sided, and p < 0.05 was considered statistically significant.

### 2.8. Machine learning analysis

This study applied supervised machine learning techniques to model the relationship between predictor variables and the binary outcome of low back pain. The analytical pipeline included data preprocessing, feature representation, model development, performance evaluation, and model interpretability. The dataset was partitioned using stratified sampling into training (80%) and testing (20%) subsets while preserving class distribution. The test set remained completely unseen during model development and was used exclusively for final model evaluation. Prior to analysis, the dataset underwent quality control procedures. Missing values were handled using appropriate imputation strategies (mean imputation for continuous variables and mode imputation for categorical variables). Categorical variables were encoded using one-hot or label encoding, while numerical variables were standardized using z-score normalization to ensure comparable feature scales and improve model convergence. Predictive modeling utilized structured input variables representing demographic, clinical, socioeconomic, and lifestyle-related characteristics that may influence the occurrence of low back pain. To prevent data leakage, all preprocessing procedures, including missing value handling and z-score standardization, were fitted using only the training data and subsequently applied to the test data. The held-out test set remained completely unseen during model development, hyperparameter optimization, and preprocessing parameter estimation. During nested cross-validation, preprocessing and class-imbalance handling were applied within the respective training folds only.

Multiple supervised learning algorithms were implemented to evaluate comparative predictive performance, including Logistic Regression (LR), Support Vector Machine with a radial basis function kernel (SVM-RBF), Random Forest (RF), Bernoulli Naïve Bayes (BNB), and Extreme Gradient Boosting (XGBoost). These algorithms represent diverse learning paradigms, including linear, probabilistic, kernel-based, and ensemble methods. Within the training dataset, model development and hyperparameter optimisation were conducted using nested stratified cross-validation with five outer folds and five inner folds. Hyperparameter optimisation was performed using RandomizedSearchCV, with ROC-AUC as the optimisation metric. RandomOverSampler was applied only within the training folds to manage class imbalance and prevent information leakage. Following model selection, final model performance was evaluated using the held-out test set. The hyperparameter search ranges and selected values are presented in the dedicated hyperparameter Table 3.

Model performance was evaluated using multiple complementary metrics. Discriminative performance was assessed using the Receiver Operating Characteristic Area Under the Curve (ROC-AUC) and Precision–Recall Area Under the Curve (PR-AUC), while classification performance was measured using accuracy, balanced accuracy, precision, recall (sensitivity), specificity, F1-score, and log loss. Confusion matrices were also examined to quantify true positives, true negatives, false positives, and false negatives, allowing detailed assessment of model prediction errors. To improve model transparency, SHapley Additive exPlanations (SHAP) analysis was conducted for the best-performing classifier to quantify the contribution of individual features to model predictions. Both global interpretability (mean absolute SHAP values) and local interpretability (instance-level explanations) were examined to better understand model decision behavior. Because the support vector machine employed a non-linear radial basis function kernel, SHAP values were computed using Kernel SHAP, a model-agnostic explainability approach. Kernel SHAP was used because a model-specific SHAP explainer is not available for a non-linear SVM-RBF model. Kernel SHAP estimates Shapley values through perturbation-based sampling and quantifies the contribution of each predictor to the model output probability. All machine learning analyses were implemented using Python.

Supplementary tables supporting the descriptive, regression, and machine-learning analyses are provided in S1 File, and the de-identified dataset used for these analyses is provided in S1 Dataset.

## 3. Results

### 3.1. Characteristics of the study participants

The final analytical sample included 566 postmenopausal women. Of these, 264 participants reported past-year low back pain, giving an overall prevalence of 46.6%. Most participants were from Dhaka division (69.1%), and the sample was almost equally distributed between urban/city (49.8%) and rural (50.2%) residence. The largest age group was 47–50 years (24.7%), followed by 51–53 years (21.2%) and 54–57 years (17.5%). Most participants were married (85.2%), Muslim (92.9%), and housewives (78.1%). Regarding health-related characteristics, 50.7% reported hypertension, 39.0% reported diabetes, 10.1% reported respiratory disease, and 11.0% reported heart disease. Calcium supplementation was reported by 69.6% of participants, whereas hormone therapy was uncommon (5.7%). Based on BMI, 50.9% were categorized as normal weight and 38.3% as overweight. Participant characteristics according to LBP status are presented in **Table 1**.

**Table 1:** Participant characteristics and bivariate associations with past-year low back pain.

Variable	Category	Total, n (%)	LBP No, n (%)	LBP Yes, n (%)	Test used	p-value	Cramer’s V
Division	Dhaka	391 (69.1%)	199 (50.9%)	192 (49.1%)	Chi-square test	0.252	0.118
	Chittagong	38 (6.7%)	23 (60.5%)	15 (39.5%)	Chi-square test	0.252	0.118
	Mymensingh	11 (1.9%)	5 (45.5%)	6 (54.5%)	Chi-square test	0.252	0.118
	Rajshahi	44 (7.8%)	27 (61.4%)	17 (38.6%)	Chi-square test	0.252	0.118
	Rangpur	11 (1.9%)	8 (72.7%)	3 (27.3%)	Chi-square test	0.252	0.118
	Khulna	31 (5.5%)	14 (45.2%)	17 (54.8%)	Chi-square test	0.252	0.118
	Barisal	40 (7.1%)	26 (65.0%)	14 (35.0%)	Chi-square test	0.252	0.118
Residence	City	282 (49.8%)	153 (54.3%)	129 (45.7%)	Chi-square test	0.669	0.018
	Rural	284 (50.2%)	149 (52.5%)	135 (47.5%)	Chi-square test	0.669	0.018
Age group	43-46	14 (2.5%)	8 (57.1%)	6 (42.9%)	Chi-square test	0.246	0.127
	47-50	140 (24.7%)	63 (45.0%)	77 (55.0%)	Chi-square test	0.246	0.127
	51-53	120 (21.2%)	73 (60.8%)	47 (39.2%)	Chi-square test	0.246	0.127
	54-57	99 (17.5%)	54 (54.5%)	45 (45.5%)	Chi-square test	0.246	0.127
	58-61	76 (13.4%)	46 (60.5%)	30 (39.5%)	Chi-square test	0.246	0.127
	62-65	80 (14.1%)	39 (48.8%)	41 (51.2%)	Chi-square test	0.246	0.127
	66-69	23 (4.1%)	12 (52.2%)	11 (47.8%)	Chi-square test	0.246	0.127
	70 and above	14 (2.5%)	7 (50.0%)	7 (50.0%)	Chi-square test	0.246	0.127
Education	Illiterate	56 (9.9%)	30 (53.6%)	26 (46.4%)	Chi-square test	0.821	0.062
	Primary	135 (23.9%)	75 (55.6%)	60 (44.4%)	Chi-square test	0.821	0.062
	Secondary	197 (34.8%)	99 (50.3%)	98 (49.7%)	Chi-square test	0.821	0.062
	Higher secondary	124 (21.9%)	67 (54.0%)	57 (46.0%)	Chi-square test	0.821	0.062
	Graduate	43 (7.6%)	26 (60.5%)	17 (39.5%)	Chi-square test	0.821	0.062
	Postgraduate	11 (1.9%)	5 (45.5%)	6 (54.5%)	Chi-square test	0.821	0.062
Marital status	Married	482 (85.2%)	259 (53.7%)	223 (46.3%)	Fisher’s exact test (simulated)	0.220	0.087
	Unmarried	7 (1.2%)	6 (85.7%)	1 (14.3%)	Fisher’s exact test (simulated)	0.220	0.087
	Widowed	74 (13.1%)	35 (47.3%)	39 (52.7%)	Fisher’s exact test (simulated)	0.220	0.087
	Divorced	3 (0.5%)	2 (66.7%)	1 (33.3%)	Fisher’s exact test (simulated)	0.220	0.087
Religion	Muslim	526 (92.9%)	278 (52.9%)	248 (47.1%)	Chi-square test	0.382	0.037
	Other	40 (7.1%)	24 (60.0%)	16 (40.0%)	Chi-square test	0.382	0.037
Occupation	Housewife	442 (78.1%)	223 (50.5%)	219 (49.5%)	Chi-square test	0.044	0.120
	Teacher	44 (7.8%)	27 (61.4%)	17 (38.6%)	Chi-square test	0.044	0.120
	Retired	41 (7.2%)	29 (70.7%)	12 (29.3%)	Chi-square test	0.044	0.120
	Other	39 (6.9%)	23 (59.0%)	16 (41.0%)	Chi-square test	0.044	0.120
Family structure	Joint family	195 (34.5%)	110 (56.4%)	85 (43.6%)	Chi-square test	0.523	0.048
	Nuclear family	360 (63.6%)	187 (51.9%)	173 (48.1%)	Chi-square test	0.523	0.048
	Alone	11 (1.9%)	5 (45.5%)	6 (54.5%)	Chi-square test	0.523	0.048
Smoking	Yes	3 (0.5%)	2 (66.7%)	1 (33.3%)	Fisher’s exact test	1.000	0.019
	No	563 (99.5%)	300 (53.3%)	263 (46.7%)	Fisher’s exact test	1.000	0.019
Sunlight exposure	Yes	298 (52.7%)	155 (52.0%)	143 (48.0%)	Chi-square test	0.499	0.028
	No	268 (47.3%)	147 (54.9%)	121 (45.1%)	Chi-square test	0.499	0.028
Hormone therapy	Yes	32 (5.7%)	20 (62.5%)	12 (37.5%)	Chi-square test	0.286	0.045
	No	534 (94.3%)	282 (52.8%)	252 (47.2%)	Chi-square test	0.286	0.045
Calcium supplementation	Yes	394 (69.6%)	200 (50.8%)	194 (49.2%)	Chi-square test	0.061	0.079
	No	172 (30.4%)	102 (59.3%)	70 (40.7%)	Chi-square test	0.061	0.079
Hypertension	Yes	287 (50.7%)	137 (47.7%)	150 (52.3%)	Chi-square test	0.007	0.114
	No	279 (49.3%)	165 (59.1%)	114 (40.9%)	Chi-square test	0.007	0.114
Diabetes	Yes	221 (39.0%)	111 (50.2%)	110 (49.8%)	Chi-square test	0.232	0.050
	No	345 (61.0%)	191 (55.4%)	154 (44.6%)	Chi-square test	0.232	0.050
Respiratory disease	Yes	57 (10.1%)	23 (40.4%)	34 (59.6%)	Chi-square test	0.038	0.087
	No	509 (89.9%)	279 (54.8%)	230 (45.2%)	Chi-square test	0.038	0.087
Heart disease	Yes	62 (11.0%)	40 (64.5%)	22 (35.5%)	Chi-square test	0.062	0.078
	No	504 (89.0%)	262 (52.0%)	242 (48.0%)	Chi-square test	0.062	0.078
Weekly exercise	Not at all	150 (26.5%)	80 (53.3%)	70 (46.7%)	Chi-square test	0.739	0.047
	1-2 times/week	230 (40.6%)	128 (55.7%)	102 (44.3%)	Chi-square test	0.739	0.047
	3-4 times/week	112 (19.8%)	58 (51.8%)	54 (48.2%)	Chi-square test	0.739	0.047
	>4 times/week	74 (13.1%)	36 (48.6%)	38 (51.4%)	Chi-square test	0.739	0.047
Abortion history	Yes	119 (21.0%)	73 (61.3%)	46 (38.7%)	Chi-square test	0.049	0.083
	No	447 (79.0%)	229 (51.2%)	218 (48.8%)	Chi-square test	0.049	0.083
BMI category	Mild thinness	8 (1.4%)	4 (50.0%)	4 (50.0%)	Fisher’s exact test (simulated)	0.725	0.059
	Normal	288 (50.9%)	156 (54.2%)	132 (45.8%)	Fisher’s exact test (simulated)	0.725	0.059
	Overweight	217 (38.3%)	110 (50.7%)	107 (49.3%)	Fisher’s exact test (simulated)	0.725	0.059
	Obese class I	49 (8.7%)	30 (61.2%)	19 (38.8%)	Fisher’s exact test (simulated)	0.725	0.059
	Obese class II	4 (0.7%)	2 (50.0%)	2 (50.0%)	Fisher’s exact test (simulated)	0.725	0.059
Socioeconomic status	Upper class	33 (5.8%)	15 (45.5%)	18 (54.5%)	Chi-square test	<0.001	0.190
	Upper middle class	271 (47.9%)	150 (55.4%)	121 (44.6%)	Chi-square test	<0.001	0.190
	Lower middle class	204 (36.0%)	92 (45.1%)	112 (54.9%)	Chi-square test	<0.001	0.190
	Upper lower class	58 (10.2%)	45 (77.6%)	13 (22.4%)	Chi-square test	<0.001	0.190

*Notes: Total percentages use the full analytic sample as the denominator. Low back pain status percentages are row percentages within each category. Fisher’s exact test was used where expected cell counts were small. Cramer’s V is reported as a measure of association strength.*

### 3.2. Associations between low back pain and participant characteristics

In bivariate analyses, occupation, hypertension, respiratory disease, abortion history, and socioeconomic status were significantly associated with past-year LBP (Table 1). The prevalence of LBP was highest among housewives (49.5%), followed by women in other occupations (41.0%), teachers (38.6%), and retired women (29.3%; p = 0.044). Participants with hypertension had a higher prevalence of LBP than those without hypertension (52.3% vs. 40.9%; p = 0.007). Similarly, participants with respiratory disease had a higher prevalence of LBP than those without respiratory disease (59.6% vs. 45.2%; p = 0.038). A history of abortion was also associated with LBP in bivariate analysis, with a lower prevalence among participants reporting abortion history than among those without abortion history (38.7% vs. 48.8%; p = 0.049). Socioeconomic status differed significantly by LBP status (p < 0.001), with the lowest prevalence observed among participants in the upper-lower socioeconomic class (22.4%).

### 3.3. Multivariable factors associated with past-year low back pain

The adjusted logistic regression results are presented in **Table 2**. The model included 566 participants and converged successfully. Model discrimination was acceptable (AUC = 0.708, 95% CI: 0.665–0.750), although McFadden’s pseudo-R² was modest (0.092) and the Hosmer–Lemeshow test suggested possible imperfect calibration (p = 0.029). Therefore, the model was interpreted as an explanatory association model rather than a clinical prediction model. Multicollinearity was not problematic, with adjusted generalized variance inflation factor values close to 1. Variable coding, model diagnostics, multicollinearity results, and full crude and adjusted estimates are provided in S1–S3 and S5 Tables in S1 File.

**Table 2:** Adjusted logistic regression analysis of factors associated with past-year low back pain.

Variable	Category	Reference	Adjusted OR (95% CI)	Adjusted p-value	Adjusted overall p-value
Division	Outside Dhaka	Outside Dhaka	Reference		0.293
	Dhaka	Outside Dhaka	1.25 (0.82, 1.91)	0.293	0.293
Residence	Rural	Rural	Reference		0.715
	City	Rural	1.08 (0.72, 1.60)	0.715	0.715
Age group	43-50	43-50	Reference		0.040
	51-57	43-50	0.50 (0.31, 0.81)	0.004	0.040
	58-65	43-50	0.61 (0.35, 1.08)	0.090	0.040
	66 and above	43-50	0.57 (0.21, 1.51)	0.256	0.040
Education	Graduate or above	Graduate or above	Reference		0.630
	Secondary or higher secondary	Graduate or above	1.17 (0.60, 2.30)	0.644	0.630
	Up to primary	Graduate or above	0.96 (0.46, 2.02)	0.920	0.630
Marital status	Not currently married	Not currently married	Reference		0.245
	Married	Not currently married	0.69 (0.37, 1.29)	0.245	0.245
Religion	Other	Other	Reference		0.189
	Muslim	Other	1.64 (0.78, 3.45)	0.193	0.189
Occupation	Other	Other	Reference		0.002
	Housewife	Other	2.20 (1.33, 3.64)	0.002	0.002
Family structure	Joint family or alone	Joint family or alone	Reference		0.786
	Nuclear family	Joint family or alone	1.06 (0.71, 1.56)	0.786	0.786
Sunlight exposure	No	No	Reference		0.053
	Yes	No	1.46 (0.99, 2.14)	0.054	0.053
Hormone therapy	No	No	Reference		0.090
	Yes	No	0.49 (0.21, 1.13)	0.095	0.090
Calcium supplementation	No	No	Reference		0.092
	Yes	No	1.44 (0.94, 2.19)	0.093	0.092
Hypertension	No	No	Reference		0.016
	Yes	No	1.60 (1.09, 2.35)	0.016	0.016
Diabetes	No	No	Reference		0.296
	Yes	No	1.23 (0.83, 1.81)	0.297	0.296
Respiratory disease	No	No	Reference		0.006
	Yes	No	2.46 (1.28, 4.71)	0.007	0.006
Heart disease	No	No	Reference		0.118
	Yes	No	0.61 (0.33, 1.14)	0.123	0.118
Weekly exercise	>4 times/week	>4 times/week	Reference		0.657
	3-4 times/week	>4 times/week	0.88 (0.46, 1.70)	0.713	0.657
	1-2 times/week	>4 times/week	0.89 (0.50, 1.61)	0.704	0.657
	Not at all	>4 times/week	1.17 (0.63, 2.18)	0.612	0.657
Abortion history	No	No	Reference		0.059
	Yes	No	0.64 (0.40, 1.02)	0.061	0.059
BMI category	Normal or thinness	Normal or thinness	Reference		0.586
	Overweight	Normal or thinness	1.21 (0.82, 1.78)	0.343	0.586
	Obese	Normal or thinness	0.94 (0.47, 1.88)	0.869	0.586
Socioeconomic status	Upper lower class	Upper lower class	Reference		<0.001
	Lower middle class	Upper lower class	3.94 (1.89, 8.23)	<0.001	<0.001
	Upper middle class	Upper lower class	3.06 (1.46, 6.39)	0.003	<0.001
	Upper class	Upper lower class	6.75 (2.31, 19.77)	<0.001	<0.001

*Notes: OR = odds ratio; CI = confidence interval. Reference categories are shown in the Reference column. Adjusted overall p-values were obtained using likelihood-ratio tests. Adjusted estimates were interpreted as associations because of the cross-sectional design.*

After adjustment, age group, occupation, hypertension, respiratory disease, and socioeconomic status were associated with past-year LBP. Compared with participants aged 43–50 years, those aged 51–57 years had lower odds of LBP (AOR = 0.50, 95% CI: 0.31–0.81, p = 0.004), while the associations for ages 58–65 years and ≥66 years were not statistically significant. Housewives had higher odds of LBP than participants in other occupational categories (AOR = 2.20, 95% CI: 1.33–3.64, p = 0.002). Higher odds of LBP were also observed among participants with hypertension (AOR = 1.60, 95% CI: 1.09–2.35, p = 0.016) and respiratory disease (AOR = 2.46, 95% CI: 1.28–4.71, p = 0.007).

Socioeconomic status was strongly associated with LBP (overall p < 0.001). Compared with participants in the upper-lower socioeconomic class, the odds of LBP were higher among those in the lower-middle class (AOR = 3.94, 95% CI: 1.89–8.23, p < 0.001), upper-middle class (AOR = 3.06, 95% CI: 1.46–6.39, p = 0.003), and upper class (AOR = 6.75, 95% CI: 2.31–19.77, p < 0.001). Sunlight exposure (AOR = 1.46, 95% CI: 0.99–2.14, p = 0.054) and abortion history (AOR = 0.64, 95% CI: 0.40–1.02, p = 0.061) did not reach statistical significance. Division, residence, education, marital status, religion, family structure, hormone therapy, calcium supplementation, diabetes, heart disease, weekly exercise, and BMI category were not significantly associated with LBP in the adjusted model.

### 3.4. Hyperparameter tuning and model selection

Hyperparameter optimization was performed using RandomizedSearchCV within the inner loop of a nested cross-validation framework (5 inner folds and 5 outer folds). The search procedure was guided by the ROC-AUC metric, and the hyperparameter search spaces and final model settings are summarized in **Table 3**. To mitigate class imbalance, RandomOverSampler was applied exclusively to the training folds during model optimization, thereby preventing information leakage into validation and held-out test data.

**Table 3:** Hyperparameter search spaces and final model settings.

Model	Hyperparameter	Search Space	Final Setting
Logistic Regression	C	Log-uniform (10 ^−^ ³–10^2^)	0.02395
	Class weight	{balanced}	balanced
	Solver	{liblinear}	liblinear
	Maximum iterations	1000	1000
Support Vector Machine (RBF)	C	Log-uniform (10 ^−^ ²–10^3^)	100.0
	Gamma (γ)	Log-uniform (10 ^−^ ⁴–10^0^)	0.35938
	Kernel	{RBF}	RBF
	Probability estimates	{True}	True
Random Forest	Number of trees (n_estimators)	100–1000	800
	Maximum depth	4–20	12
	Minimum samples split	2–20	2
	Minimum samples leaf	1–10	1
	Class weight	{balanced, balanced_subsample}	balanced_subsample
Bernoulli Naïve Bayes	Smoothing parameter (α)	Log-uniform (10 ^−^ ²–10^1^)	7.279
XGBoost	Number of trees (n_estimators)	100–1000	Selected through RandomizedSearchCV
	Learning rate	0.01–0.30	Selected through RandomizedSearchCV
	Maximum depth	3–10	Selected through RandomizedSearchCV
	Subsample	0.6–1.0	Selected through RandomizedSearchCV
	Column sample by tree	0.6–1.0	Selected through RandomizedSearchCV
	Objective function	Binary logistic	Binary logistic
	Tree method	GPU Hist	GPU Hist

*Note: RandomizedSearchCV was conducted within the inner cross-validation loop using ROC-AUC as the optimization metric.*

### 3.5. SHAP-Based Model Interpretability Analysis

**Fig 1:**
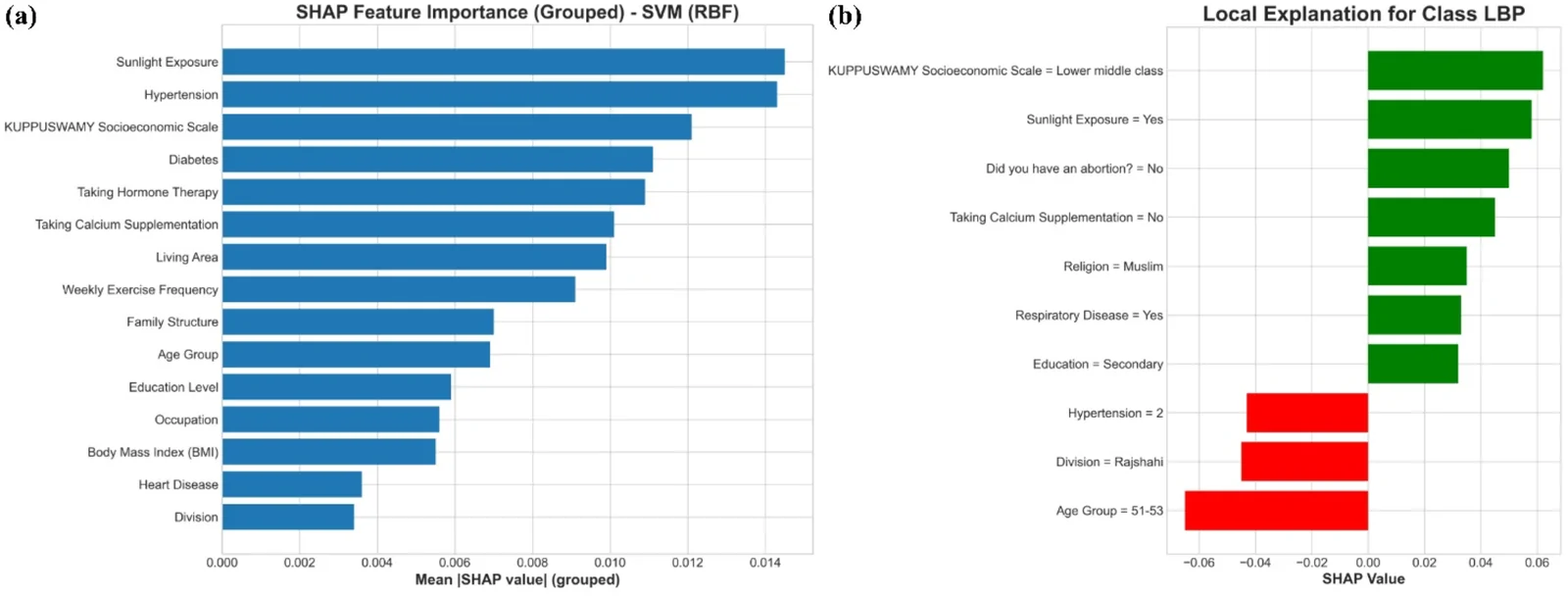
SHAP explanations for the SVM-RBF classifier. (a) Global feature importance derived from grouped mean absolute SHAP values. (b) Local SHAP explanation for one correctly classified LBP-positive case from the held-out test set. Positive SHAP values indicate features contributing toward the predicted LBP classification, whereas negative values indicate features opposing the prediction.

SHapley Additive exPlanations (SHAP) analysis was performed for the best-performing classifier, the Support Vector Machine with radial basis function (SVM-RBF), to examine feature contributions to model predictions. Fig 1 presents both global and local SHAP interpretations.

The grouped SHAP feature importance values in Fig 1(a) indicate that sunlight exposure had the largest grouped mean absolute SHAP value, followed by hypertension and socioeconomic status. Other variables with relatively high importance included diabetes, hormone therapy use, and calcium supplementation. Variables such as weekly exercise frequency, living area, and family structure showed moderate importance, while age group, education level, occupation, body mass index, heart disease, and division contributed comparatively less to model predictions.

Fig 1(b) presents a local SHAP explanation for one correctly classified past-year LBP-positive case from the held-out test set. Positive SHAP values (green bars) indicate features increasing the predicted probability of LBP, while negative values (red bars) indicate features decreasing it. For this instance, positive contributions were observed for lower-middle socioeconomic status, sunlight exposure, no abortion history, no calcium supplementation, Muslim religion, respiratory disease, and secondary education level. Negative contributions were associated with age group 51–53, Rajshahi division, and hypertension. These feature contributions relate to this individual prediction and should not be interpreted as population-level associations.

### 3.6. Cross-Validation Performance of Machine Learning Models

The classification performance of the evaluated machine learning models obtained during nested cross-validation is summarized in **Table 4**. Values are reported as mean ± standard deviation across the outer cross-validation folds. Among the models, the SVM-RBF classifier achieved the highest overall accuracy (0.772 ± 0.030) and precision (0.792 ± 0.051). It also produced the highest specificity (0.834 ± 0.059) and the lowest log loss (0.438 ± 0.030). The Random Forest model achieved comparable performance and recorded the highest F1-score (0.744 ± 0.040), with sensitivity of 0.726 ± 0.061 and specificity of 0.805 ± 0.061. The XGBoost classifier showed moderate performance with the highest recall (0.745 ± 0.127) but lower specificity compared with SVM-RBF and Random Forest. In contrast, Logistic Regression and Bernoulli Naïve Bayes showed lower performance across most evaluation metrics. Logistic Regression achieved an accuracy of 0.564 ± 0.037, while Bernoulli Naïve Bayes achieved 0.549 ± 0.029.

**Table 4:** Cross-validation performance of machine learning models in the training data. Values are reported as mean ± standard deviation across the outer folds.

Model	Accuracy	Precision	Recall (Sensitivity)	Specificity	F1-Score	Log Loss
SVM (RBF)	0.772 ± 0.030	0.792 ± 0.051	0.702 ± 0.088	0.834 ± 0.059	0.740 ± 0.043	0.438 ± 0.030
Random Forest	0.768 ± 0.035	0.768 ± 0.055	0.726 ± 0.061	0.805 ± 0.061	0.744 ± 0.040	0.508 ± 0.018
XGBoost (GPU)	0.744 ± 0.066	0.715 ± 0.057	0.745 ± 0.127	0.742 ± 0.056	0.727 ± 0.086	0.721 ± 0.133
Logistic Regression	0.564 ± 0.037	0.533 ± 0.040	0.560 ± 0.050	0.569 ± 0.065	0.545 ± 0.037	0.715 ± 0.060
Bernoulli Naïve Bayes	0.549 ± 0.029	0.514 ± 0.028	0.597 ± 0.045	0.506 ± 0.034	0.552 ± 0.032	0.761 ± 0.053

### 3.7. Discriminative Performance on the Held-Out Test Set

**Fig 2:**
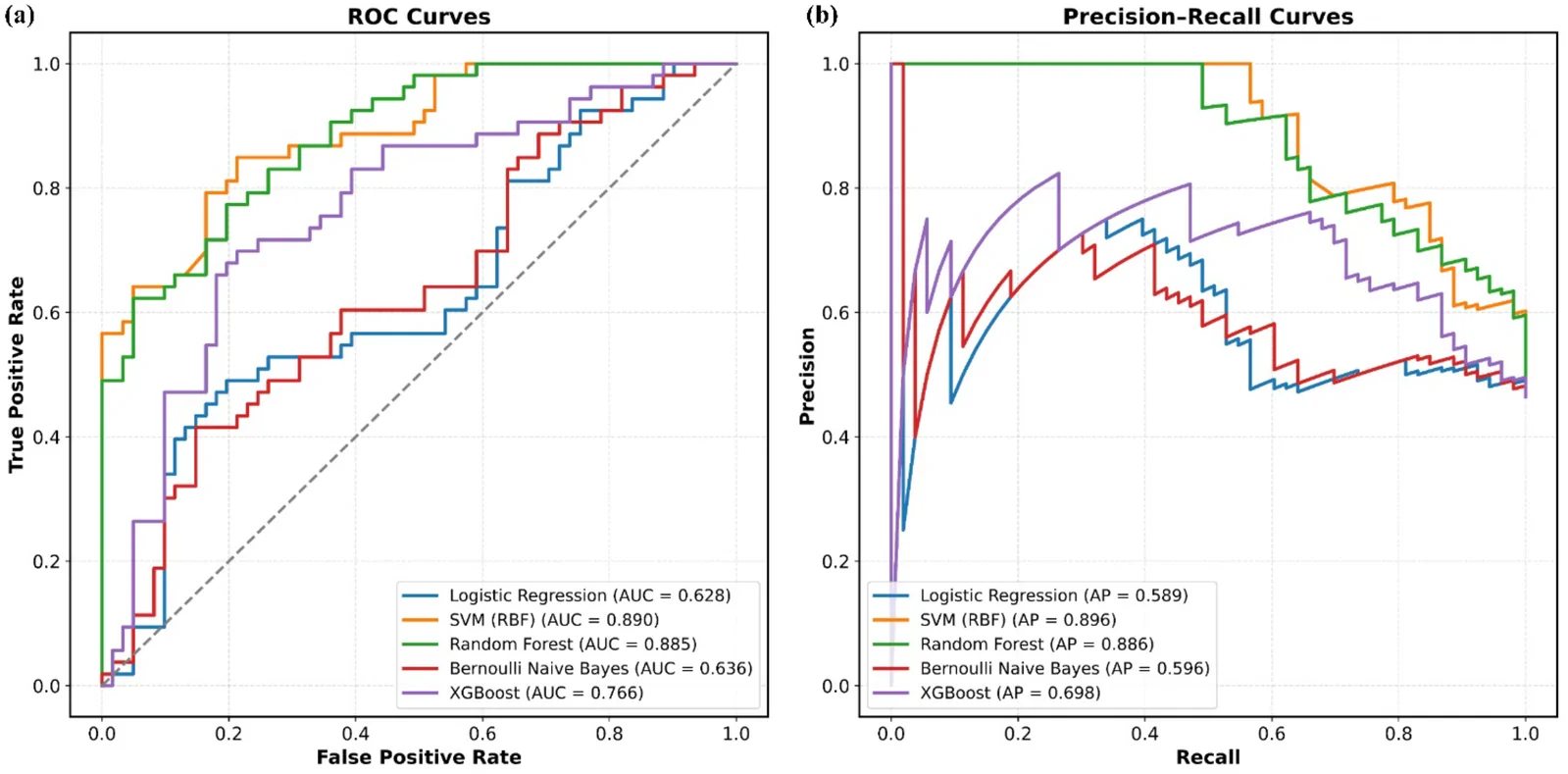
Discriminative performance of machine learning classifiers on the held-out test set. (a) Receiver Operating Characteristic (ROC) curves. (b) Precision–Recall (PR) curves.

The discriminative ability of the classifiers was evaluated using Receiver Operating Characteristic (ROC) and Precision–Recall (PR) analyses on the held-out test set (Fig 2). The ROC curves in Fig 2(a) show that the SVM-RBF model achieved the highest ROC-AUC value (0.890), followed closely by the Random Forest model (0.885). The XGBoost classifier achieved a ROC-AUC of 0.766, while Logistic Regression and Bernoulli Naïve Bayes showed lower values of 0.628 and 0.636, respectively. The Precision–Recall curves shown in Fig 2(b) demonstrate similar patterns. The SVM-RBF model achieved the highest average precision (AP = 0.896), followed by Random Forest (AP = 0.886). The XGBoost model achieved an AP value of 0.698, whereas Logistic Regression and Bernoulli Naïve Bayes recorded lower values of 0.589 and 0.596, respectively.

### 3.8. Classification Error Analysis on the Held-Out Test Set

**Fig 3:**
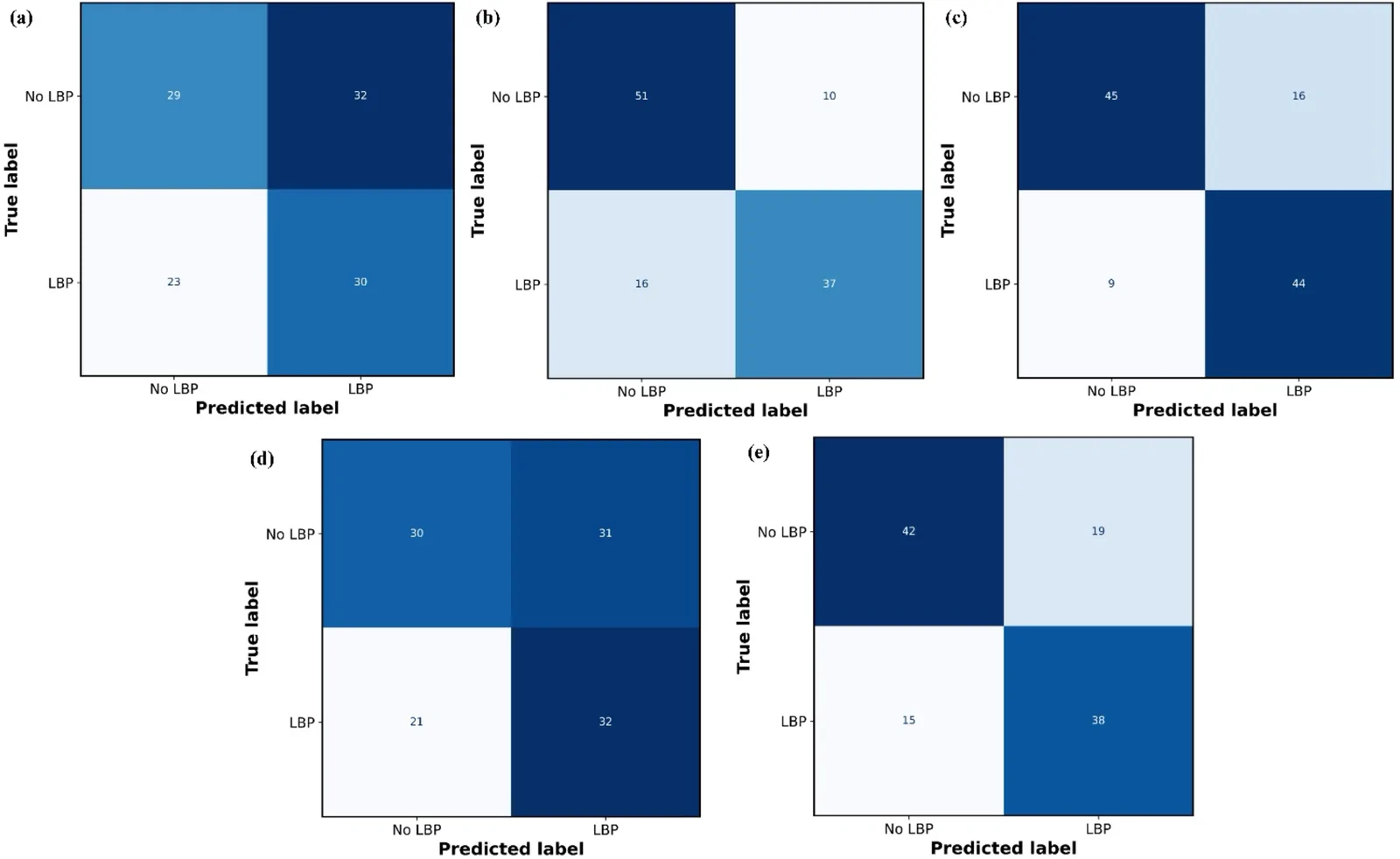
Confusion matrices of machine learning classifiers evaluated on the held-out test set. (a) Logistic Regression, (b) Support Vector Machine with radial basis function (SVM-RBF), (c) Random Forest, (d) Bernoulli Naïve Bayes, and (e) XGBoost.

Confusion matrices for all classifiers were evaluated on the held-out test set (Fig 3). The SVM-RBF classifier correctly identified 51 non-LBP cases (true negatives) and 37 LBP cases (true positives). The Random Forest model produced 44 true positives and 9 false negatives, indicating greater sensitivity for identifying LBP cases than the SVM-RBF model. The XGBoost classifier correctly predicted 38 true positives but produced a slightly higher number of false positives compared with SVM-RBF and Random Forest. In contrast, Logistic Regression and Bernoulli Naïve Bayes showed higher numbers of both false positives and false negatives. Across the evaluated models, SVM-RBF and Random Forest showed the lowest overall misclassification rates, whereas Logistic Regression and Bernoulli Naïve Bayes demonstrated comparatively higher classification errors.

## 4. Discussion

This clinic-based cross-sectional study found that past-year LBP was reported by 46.6% of postmenopausal women attending selected rehabilitation and physiotherapy facilities in Bangladesh. According to the Global Burden of Disease Study, LBP remains the single greatest contributor to years lived with disability worldwide, with the number of affected people expected to increase in tandem with population aging [7]. While LBP is widely studied in high-income settings, the specific vulnerability of postmenopausal women in low- and middle-income countries such as Bangladesh has been understudied. The present findings contribute evidence from a healthcare-seeking population in Bangladesh and highlight the need for further context-specific research.

Our observed prevalence is higher than the national average reported by Majumder et al. [15], who found a weighted prevalence of 18.5% among Bangladeshi adults and an age-standardized prevalence of 27.2% among women, but falls within the range of findings from other Asian and international samples of postmenopausal women. For instance, a Nigerian study reported a 12-month prevalence of back symptoms of 52.9%, while a Northern Indian study reported LBP prevalence of 77.8% [11,13], while Turkish postmenopausal women have been found to experience even higher rates, up to 90.2% [12]. Similarly, iin an Italian trial, approximately one-third of healthy postmenopausal women reported LBP at baseline, with no statistically significant between-group difference in LBP prevalence following the physical activity intervention [14]. However, these estimates should not be interpreted as directly comparable because they arise from different study designs, sampling frames, LBP definitions, recall periods, and healthcare settings. The relatively high prevalence in the present study may partly reflect recruitment from rehabilitation centers among women seeking care for musculoskeletal complaints. Therefore, the 46.6% estimate should be interpreted as a prevalence within this sampled clinical population, rather than as a nationwide community estimate.

In the adjusted analysis, socioeconomic status and occupation emerged as important social-context factors associated with past-year LBP. Compared with women in the upper-lower socioeconomic class, women in the lower-middle, upper-middle, and upper classes had higher odds of LBP. This association is consistent with the social determinants of health framework, which posits that income, education, and occupation influence both exposure to risk factors and vulnerability to musculoskeletal conditions [1,22]. However, the socioeconomic status measure used in this study combines education, occupation, and household income, and the observed association may reflect differences in social position, healthcare-seeking patterns, access to rehabilitation services, domestic work roles, and other unmeasured exposures [21]. The higher odds of LBP among housewives may also reflect the physical demands of household work, including prolonged standing, repetitive bending, lifting, floor-level activities, caregiving responsibilities, and limited opportunity for structured rest or ergonomic adjustment. In many South Asian settings, domestic responsibilities may involve sustained musculoskeletal load that is not captured by formal employment categories. Because detailed information on household activities, lifting, working posture, caregiving, and other ergonomic exposures was not collected, the interplay between socioeconomic status, work type, healthcare access, and LBP warrants further investigation in studies that directly assess daily physical demands and work-related exposures.

The study also identified adjusted associations of hypertension and respiratory disease with past-year LBP. However, Bae et al. reported an inverse adjusted association between hypertension and LBP in Korean adults [23], indicating that findings differ across populations. Vascular complications in diabetes and disc degeneration in postmenopausal women have been described; however, their relevance to the association observed in the present study remains uncertain [24,25]. The biopsychosocial approach suggests that LBP management should be considered within the broader framework of biological, psychological, and social factors [1,3]. Respiratory disease was also associated with higher odds of past-year LBP, echoing evidence linking conditions such as COPD and asthma to increased risk of back pain [26]. Chronic coughing, altered trunk biomechanics, thoracic restriction, respiratory muscle dysfunction, and reduced physical capacity may increase mechanical strain on the spine, while shared environmental exposures and chronic inflammation may also contribute to this association [27]. The mechanisms underlying this association remain uncertain because the cross-sectional design does not establish temporal ordering, and the present study did not assess respiratory disease severity, lung function, medication use, or physical performance.

Heart disease and BMI category were not significantly associated with past-year LBP in the adjusted model. Therefore, the present findings do not support an independent association between heart disease and LBP in this sample. Similarly, although higher BMI has been investigated in relation to spinal and pelvic alignment [28], BMI category was not clearly associated with LBP in our analysis. Yoo et al. [29] reported associations between body composition and musculoskeletal pain in women, suggesting that BMI alone may not capture all relevant aspects of body composition. The lack of clear evidence of an association may reflect the clinic-based sampling frame, categorical BMI grouping, sparse numbers in some BMI categories, or the inability of BMI to capture central adiposity, body composition, muscle strength, and functional capacity. Sunlight exposure showed a borderline but non-significant association with past-year LBP. Because sunlight exposure was self-reported and no vitamin D measurements, occupational exposure data, or detailed outdoor activity information were available, this finding should be interpreted cautiously. Hormone therapy was not significantly associated with LBP, and abortion history did not remain statistically significant after adjustment. These findings should therefore be interpreted as inconclusive and should not be taken as evidence of hormonal, reproductive, or psychosocial mechanisms [6].

Our machine learning analysis showed that nonlinear models, particularly support vector machine and random forest models, achieved better internal classification performance than traditional linear approaches. This finding is consistent with previous studies showing that machine learning methods can support classification and outcome prediction in chronic low back pain by capturing complex relationships among demographic, clinical, functional, and psychosocial factors. For example, ensemble and tree-based models have been used to predict pain intensity, disability, treatment outcomes, and prognostic profiles among patients with chronic or subacute low back pain [30–32]. In addition, population-based research has shown that artificial neural networks and other machine learning models can model patterns associated with chronic low back pain by capturing complex relationships among demographic and health-related variables [33]. To reduce overfitting, model development used nested cross-validation, held-out test-set evaluation, and model-specific regularization. Nevertheless, the machine learning findings should be interpreted as internally evaluated classification results rather than evidence of clinical prediction utility. Several features identified as important through SHAP analysis, including hypertension and socioeconomic status, were also associated with LBP in the adjusted logistic regression analysis. However, some variables with high SHAP importance were not statistically significant in the regression model, likely reflecting differences between model-specific feature contribution and regression-based adjusted association. Nonlinear models such as SVM-RBF may capture interactions and nonlinear patterns that are not represented in conventional logistic regression. Therefore, while logistic regression provides interpretable estimates of adjusted associations, machine learning models may offer complementary insights into multifactorial patterns underlying LBP classification. SHAP values should be interpreted as model-specific contributions to prediction, not as causal importance.

Taken together, these findings reinforce that past-year LBP among postmenopausal women attending rehabilitation and physiotherapy facilities in Bangladesh is multifactorial, reflecting the interplay of comorbid health conditions, socioeconomic circumstances, occupational and household roles, and other factors not fully captured in the present study. The biopsychosocial model of pain is particularly relevant in this context, underscoring the need for integrated approaches to musculoskeletal health, chronic disease management, and rehabilitation [1,3]. In addition to conventional epidemiological approaches, machine learning techniques may offer useful tools for examining LBP classification patterns by integrating multiple demographic, clinical, socioeconomic, and lifestyle factors simultaneously. However, such models require external validation, calibration assessment, and evaluation of clinical utility before they can be considered for use in primary care, rehabilitation, or community settings. Public health and clinical strategies may benefit from ergonomic education, improved access to rehabilitation services, and integrated management of musculoskeletal symptoms and chronic conditions. However, the present findings do not establish the effectiveness of these strategies, and prospective studies are needed to assess their potential value.

### 4.1. Strengths and limitations

This study has several strengths. First, it focuses on a moderately sized sample of postmenopausal women, an under-researched group in Bangladesh, and provides an assessment of sociodemographic, health-related, lifestyle, BMI, and socioeconomic factors associated with past-year LBP. The inclusion of diverse demographic, clinical, socioeconomic, and lifestyle variables allowed a broader evaluation of the multifactorial nature of LBP in this healthcare-seeking population. In addition, the integration of machine learning techniques alongside conventional statistical analyses provided complementary insights into LBP classification patterns and model-specific feature contributions.

However, several limitations should be acknowledged. The cross-sectional design precludes conclusions regarding causality or the temporal direction of the observed associations. Additionally, because this study focused entirely on postmenopausal women visiting selected rehabilitation and physiotherapy facilities, the 46.6% prevalence estimate reflects a specific healthcare-seeking clinical population. This estimate should be interpreted within a clinical context rather than as a general nationwide community estimate. Participants were recruited from a limited number of facilities using a facility-based sampling approach, which may have introduced selection bias and further limits generalizability to postmenopausal women who do not seek rehabilitation or physiotherapy care.

Furthermore, while a structured questionnaire format was the most practical method for this field-based survey, self-reported information on sunlight exposure, exercise, supplementation use, comorbidity status, reproductive history, and LBP status may have been affected by recall or reporting error. LBP was assessed using structured self-report rather than clinical examination or imaging, and information on pain severity, duration, frequency, disability, and treatment history was not collected. Reported low-back diagnoses were recorded where available, but underlying spinal pathology was not independently verified by clinical examination or imaging. In contrast, height and weight were measured, although BMI may still not capture central adiposity, body composition, muscle strength, or functional capacity. There is also the possibility of residual confounding due to unmeasured factors, including psychosocial stress, depression, anxiety, sleep quality, detailed physical activity patterns, dietary behaviors, occupational and household ergonomic exposures, medication use, objective cardiometabolic markers, vitamin D status, bone mineral density, and physical performance measures that were not assessed in this study. Furthermore, some variables were categorized for analysis, which may have reduced information and statistical power.

Although machine learning models demonstrated internal classification performance, their results should be interpreted cautiously because model performance may vary across different populations and settings. Another limitation of this study is the absence of external validation using independent datasets from different populations or institutions. Although the models were evaluated using a held-out test set, further validation in external cohorts is necessary before broader application can be considered. Although rigorous validation procedures were employed, including nested cross-validation and held-out test-set evaluation, the sample size remains relatively modest for machine learning applications, particularly given the number of candidate predictors, which may raise the possibility of model instability or optimistic performance estimates. Therefore, the findings should be interpreted with caution until validated in larger, independent, and multicenter populations. Finally, the absence of longitudinal follow-up limits the ability to examine changes in LBP status over time, assess incidence or persistence of symptoms, or evaluate the long-term impact of potential preventive or rehabilitative interventions.

## 5. Conclusions

This study found that past-year low back pain was commonly reported among postmenopausal women attending selected rehabilitation and physiotherapy facilities in Bangladesh. In adjusted analyses, age group, occupation, hypertension, respiratory disease, and socioeconomic status were associated with past-year LBP, whereas BMI, diabetes, heart disease, weekly exercise, hormone therapy, and calcium supplementation were not clearly associated. These findings suggest that LBP in this healthcare-seeking population may reflect a complex interplay of social, occupational, and health-related factors. The results support further evaluation of integrated approaches to musculoskeletal symptoms and chronic-condition management among women seeking rehabilitation care. However, the study does not establish causality or the effectiveness of specific preventive or rehabilitative strategies, and the findings should not be generalized to postmenopausal women in the wider Bangladeshi community. Future longitudinal community- and clinic-based studies with detailed ergonomic, psychosocial, and objective health measures are needed to clarify temporal relationships and confirm these findings. External validation is also required before the machine learning models can be considered for broader clinical use.

## Supporting information

S1 File Supplementary tables supporting the descriptive, regression, and machine-learning analyses. (DOCX)

S1 Dataset De-identified dataset used for the statistical and machine-learning analyses. (XLSX)
