# Supplementary material for: Prevalence, associated factors, and machine learning classification of low back pain among postmenopausal women attending rehabilitation and physiotherapy facilities in Bangladesh
Source: PLoS One. 2026 Sep 21;21(9):e0355864. doi: 10.1371/journal.pone.0355864 (PMC13592641; doi:10.1371/journal.pone.0355864)
Supplement: Supplementary file 1 [file pone.0355864.s001.docx]

# Supplementary File

# *Prevalence, associated factors, and machine learning classification of low back pain among postmenopausal women attending rehabilitation and physiotherapy facilities in Bangladesh*

## Outcome definition

The primary outcome was previous-12-month low back pain, coded as 1 = Yes and 0 = No.

## Dataset summary

| Item | Value |
| --- | --- |
| Total analytic sample | 566 |
| LBP = Yes | 264 |
| LBP = No | 302 |
| Missing outcome values | 0 |
| Smoking exposed participants | 3 |

## Supplementary Table S1. Coding of variables used in the descriptive, regression, and machine-learning analyses.

| variable | descriptive_table_categories | adjusted_regression_categories | reason |
| --- | --- | --- | --- |
| Division | Original categories | Dhaka vs outside Dhaka | Several non-Dhaka divisions had small cell counts. |
| Age group | Original categories | 43-50, 51-57, 58-65, 66 and above | Original extreme age categories were sparse. |
| Education | Original categories | Graduate or above, secondary or higher secondary, up to primary | Higher education categories were sparse. |
| Marital status | Original categories | Married vs not currently married | Unmarried and divorced categories were sparse. |
| Occupation | Original categories | Housewife vs other | Non-housewife categories were smaller. |
| Family structure | Original categories | Nuclear family vs joint family or alone | Alone category was sparse. |
| BMI category | Original categories | Normal/thinness, overweight, obese | Thinness and obese class II categories were sparse. |
| Smoking | Original categories | Not included | Sparse exposure count. |

## Supplementary Table S2. Diagnostic statistics for the adjusted logistic regression model.

| Item | Value |
| --- | --- |
| Regression sample size | 566 |
| Events: LBP Yes | 264 |
| Non-events: LBP No | 302 |
| Number of model parameters excluding intercept | 27 |
| Events per parameter using smaller outcome group | 9.78 |
| Model converged | TRUE |
| AIC | 766.11 |
| BIC | 887.59 |
| McFadden pseudo-R² | 0.092 |
| AUC | 0.708 |
| AUC 95% CI | 0.665, 0.750 |
| Hosmer-Lemeshow p-value | 0.029 |

## Supplementary Table S3. Multicollinearity assessment for the adjusted logistic regression model.

| Variable | GVIF | Df | Adjusted GVIF |
| --- | --- | --- | --- |
| Division | 1.192 | 1 | 1.092 |
| Residence | 1.260 | 1 | 1.123 |
| Age group | 2.414 | 3 | 1.158 |
| Education | 1.525 | 2 | 1.111 |
| Marital status | 1.573 | 1 | 1.254 |
| Religion | 1.104 | 1 | 1.051 |
| Occupation | 1.362 | 1 | 1.167 |
| Family structure | 1.136 | 1 | 1.066 |
| Sunlight exposure | 1.184 | 1 | 1.088 |
| Hormone therapy | 1.194 | 1 | 1.093 |
| Calcium supplementation | 1.199 | 1 | 1.095 |
| Hypertension | 1.186 | 1 | 1.089 |
| Diabetes | 1.168 | 1 | 1.081 |
| Respiratory disease | 1.211 | 1 | 1.100 |
| Heart disease | 1.102 | 1 | 1.050 |
| Weekly exercise | 1.433 | 3 | 1.062 |
| Abortion history | 1.149 | 1 | 1.072 |
| BMI category | 1.274 | 2 | 1.062 |
| Socioeconomic status | 1.656 | 3 | 1.088 |

Notes: For multi-level categorical predictors, adjusted GVIF is reported as GVIF^(1/(2 × Df)).

## Supplementary Table S4. Sparse original categories considered during variable recoding and model development.

| variable | category | n | percent |
| --- | --- | --- | --- |
| bmi_category | Obese class II | 4 | 0.7 |
| bmi_category | Mild thinness | 8 | 1.4 |
| marital_status | Divorced | 3 | 0.5 |
| marital_status | Unmarried | 7 | 1.2 |
| smoking | Yes | 3 | 0.5 |

## Supplementary Table S5. Crude and adjusted logistic regression estimates for factors associated with past-year low back pain.

| Variable | Category | Reference | Crude OR (95% CI) | Crude p-value | Crude overall p-value | Adjusted OR (95% CI) | Adjusted p-value | Adjusted overall p-value |
| --- | --- | --- | --- | --- | --- | --- | --- | --- |
| Division | Outside Dhaka | Outside Dhaka | Reference |  | 0.079 | Reference |  | 0.293 |
| Division | Dhaka | Outside Dhaka | 1.38 (0.96, 1.98) | 0.080 | 0.079 | 1.25 (0.82, 1.91) | 0.293 | 0.293 |
| Residence | Rural | Rural | Reference |  | 0.669 | Reference |  | 0.715 |
| Residence | City | Rural | 0.93 (0.67, 1.29) | 0.669 | 0.669 | 1.08 (0.72, 1.60) | 0.715 | 0.715 |
| Age group | 43-50 | 43-50 | Reference |  | 0.152 | Reference |  | 0.040 |
| Age group | 51-57 | 43-50 | 0.62 (0.41, 0.94) | 0.024 | 0.152 | 0.50 (0.31, 0.81) | 0.004 | 0.040 |
| Age group | 58-65 | 43-50 | 0.71 (0.46, 1.12) | 0.140 | 0.152 | 0.61 (0.35, 1.08) | 0.090 | 0.040 |
| Age group | 66 and above | 43-50 | 0.81 (0.40, 1.66) | 0.566 | 0.152 | 0.57 (0.21, 1.51) | 0.256 | 0.040 |
| Education | Graduate or above | Graduate or above | Reference |  | 0.636 | Reference |  | 0.630 |
| Education | Secondary or higher secondary | Graduate or above | 1.26 (0.70, 2.25) | 0.439 | 0.636 | 1.17 (0.60, 2.30) | 0.644 | 0.630 |
| Education | Up to primary | Graduate or above | 1.10 (0.60, 2.03) | 0.751 | 0.636 | 0.96 (0.46, 2.02) | 0.920 | 0.630 |
| Marital status | Not currently married | Not currently married | Reference |  | 0.666 | Reference |  | 0.245 |
| Marital status | Married | Not currently married | 0.90 (0.57, 1.44) | 0.666 | 0.666 | 0.69 (0.37, 1.29) | 0.245 | 0.245 |
| Religion | Other | Other | Reference |  | 0.380 | Reference |  | 0.189 |
| Religion | Muslim | Other | 1.34 (0.69, 2.58) | 0.384 | 0.380 | 1.64 (0.78, 3.45) | 0.193 | 0.189 |
| Occupation | Other | Other | Reference |  | 0.008 | Reference |  | 0.002 |
| Occupation | Housewife | Other | 1.72 (1.14, 2.60) | 0.009 | 0.008 | 2.20 (1.33, 3.64) | 0.002 | 0.002 |
| Family structure | Joint family or alone | Joint family or alone | Reference |  | 0.373 | Reference |  | 0.786 |
| Family structure | Nuclear family | Joint family or alone | 1.17 (0.83, 1.65) | 0.373 | 0.373 | 1.06 (0.71, 1.56) | 0.786 | 0.786 |
| Sunlight exposure | No | No | Reference |  | 0.499 | Reference |  | 0.053 |
| Sunlight exposure | Yes | No | 1.12 (0.81, 1.56) | 0.499 | 0.499 | 1.46 (0.99, 2.14) | 0.054 | 0.053 |
| Hormone therapy | No | No | Reference |  | 0.283 | Reference |  | 0.090 |
| Hormone therapy | Yes | No | 0.67 (0.32, 1.40) | 0.288 | 0.283 | 0.49 (0.21, 1.13) | 0.095 | 0.090 |
| Calcium supplementation | No | No | Reference |  | 0.060 | Reference |  | 0.092 |
| Calcium supplementation | Yes | No | 1.41 (0.98, 2.03) | 0.062 | 0.060 | 1.44 (0.94, 2.19) | 0.093 | 0.092 |
| Hypertension | No | No | Reference |  | 0.006 | Reference |  | 0.016 |
| Hypertension | Yes | No | 1.58 (1.14, 2.21) | 0.007 | 0.006 | 1.60 (1.09, 2.35) | 0.016 | 0.016 |
| Diabetes | No | No | Reference |  | 0.232 | Reference |  | 0.296 |
| Diabetes | Yes | No | 1.23 (0.88, 1.72) | 0.232 | 0.232 | 1.23 (0.83, 1.81) | 0.297 | 0.296 |
| Respiratory disease | No | No | Reference |  | 0.038 | Reference |  | 0.006 |
| Respiratory disease | Yes | No | 1.79 (1.03, 3.13) | 0.040 | 0.038 | 2.46 (1.28, 4.71) | 0.007 | 0.006 |
| Heart disease | No | No | Reference |  | 0.060 | Reference |  | 0.118 |
| Heart disease | Yes | No | 0.60 (0.34, 1.03) | 0.064 | 0.060 | 0.61 (0.33, 1.14) | 0.123 | 0.118 |
| Weekly exercise | >4 times/week | >4 times/week | Reference |  | 0.740 | Reference |  | 0.657 |
| Weekly exercise | 3-4 times/week | >4 times/week | 0.88 (0.49, 1.59) | 0.675 | 0.740 | 0.88 (0.46, 1.70) | 0.713 | 0.657 |
| Weekly exercise | 1-2 times/week | >4 times/week | 0.75 (0.45, 1.28) | 0.294 | 0.740 | 0.89 (0.50, 1.61) | 0.704 | 0.657 |
| Weekly exercise | Not at all | >4 times/week | 0.83 (0.47, 1.45) | 0.509 | 0.740 | 1.17 (0.63, 2.18) | 0.612 | 0.657 |
| Abortion history | No | No | Reference |  | 0.048 | Reference |  | 0.059 |
| Abortion history | Yes | No | 0.66 (0.44, 1.00) | 0.050 | 0.048 | 0.64 (0.40, 1.02) | 0.061 | 0.059 |
| BMI category | Normal or thinness | Normal or thinness | Reference |  | 0.420 | Reference |  | 0.586 |
| BMI category | Overweight | Normal or thinness | 1.14 (0.81, 1.63) | 0.451 | 0.420 | 1.21 (0.82, 1.78) | 0.343 | 0.586 |
| BMI category | Obese | Normal or thinness | 0.77 (0.43, 1.40) | 0.395 | 0.420 | 0.94 (0.47, 1.88) | 0.869 | 0.586 |
| Socioeconomic status | Upper lower class | Upper lower class | Reference |  | <0.001 | Reference |  | <0.001 |
| Socioeconomic status | Lower middle class | Upper lower class | 4.21 (2.14, 8.28) | <0.001 | <0.001 | 3.94 (1.89, 8.23) | <0.001 | <0.001 |
| Socioeconomic status | Upper middle class | Upper lower class | 2.79 (1.44, 5.41) | 0.002 | <0.001 | 3.06 (1.46, 6.39) | 0.003 | <0.001 |
| Socioeconomic status | Upper class | Upper lower class | 4.15 (1.65, 10.45) | 0.002 | <0.001 | 6.75 (2.31, 19.77) | <0.001 | <0.001 |
